# Outcomes of surgery for patients with Behcet’s disease causing aortic pseudoaneurysm: a shift from open surgery to endovascular repair

**DOI:** 10.6061/clinics/2016(06)03

**Published:** 2016-06

**Authors:** Chenyang Shen, Weihao Li, Yongbao Zhang, Qingle Li, Yang Jiao, Tao Zhang, Xiaoming Zhang

**Affiliations:** I Chinese Academy of Medical Sciences and Peking Union Medical College, Fuwai Hospital, National Center for Cardiovascular Diseases, Center of Vascular Surgery, Beijing, China; II Peking University People’s Hospital, Department of Vascular Surgery, Beijing, China

**Keywords:** Aneurysm, False, Aortic Aneurysm, Behcet’s Disease, Vascular Surgical Procedures

## Abstract

**OBJECTIVES:**

Behcet’s disease is a form of systematic vasculitis that affects vessels of various sizes. Aortic pseudoaneurysm is one of the most important causes of death among patients with Behcet’s disease due to its high risk of rupture and associated mortality. Our study aimed to investigate the outcomes of Behcet’s disease patients with aortic pseudoaneurysms undergoing open surgery and endovascular aortic repair.

**METHODS:**

From January 2003 to September 2014, ten consecutive patients undergoing surgery for aortic pseudoaneurysm met the diagnostic criteria for Behcet’s disease. Endovascular repair was the preferred modality and open surgery was performed as an alternative. Systemic immunosuppressive medication was administered after Behcet’s disease was definitively diagnosed.

**RESULTS:**

Eight patients initially underwent endovascular repair and two patients initially underwent open surgery. The overall success rate was 90% and the only failed case involved the use of the chimney technique to reach a suprarenal location. The median follow-up duration was 23 months. There were 7 recurrences in 5 patients. The median interval between operation and recurrence was 13 months. No significant risk factors for recurrence were identified, but a difference in recurrence between treatment and non-treatment with preoperative immunosuppressive medication preoperatively was notable. Four aneurysm-related deaths occurred within the follow-up period. The overall 1-year, 3-year and 5-year survival rates were 80%, 64% and 48%, respectively.

**CONCLUSIONS:**

Both open surgery and endovascular repair are safe and effective for treating aortic pseudoaneurysm in Behcet’s disease patients. The results from our retrospective study indicated that immunosuppressive medication was essential to defer the occurrence and development of recurrent aneurysms.

## INTRODUCTION

Behcet’s disease (BD) is particularly common in regions along the Silk Road from the Mediterranean to the Far East. This disease manifests as oral and genital ulcerations, skin lesions, uveitis and vascular, central nervous system and gastrointestinal involvement 1. As BD is a systemic vasculitis of small and large vessels, including both veins and arteries, large vascular damage in BD patients, termed vasculo-BD, is characterized by venous thrombosis, arterial occlusion and aneurysm. Vasculo-BD has been reported to occur in 14.7% - 27.7% of BD patients 2,3. Arterial involvement has drawn special attention due to its significant association with mortality, as this complication accounts for over a quarter of all BD-related deaths 4. Although pulmonary arterial aneurysm is much more common in BD patients than in the general population, aortic aneurysm remains one of the principal causes of death due its high risk of rupture and associated mortality 5.

Surgical repair combined with systemic immunosuppressant administration has been recommended for peripheral arterial aneurysm and this recommendation is primarily supported by experience and evidence from open trials and observational studies 6. Surgical bypass was formerly the traditional choice, and its safety and effectiveness for enabling survival of aneurysmal rupture have been confirmed. However, anastomotic pseudoaneurysm is a major adverse event. Hosaka reported that the overall 5-year cumulative incidence of anastomotic pseudoaneurysm formation after open surgery was 12.9% 7.

Endovascular aortic repair (EVAR) is minimally invasive, is completed in a short operative time and does not require general anesthesia. This method has been demonstrated to be an ideal urgent operative modality for patients with a ruptured abdominal aortic aneurysm (AAA) 8,9. Moreover, the introduction of EVAR should reduce the recurrence of pseudoaneurysms at arterial anastomoses in BD patients 10. Several studies have focused on the efficacy and safety of endovascular treatment for vasculo-BD patients, but clinical evidence indicating the prognosis of BD patients with aortic pseudoaneurysms after EVAR remains lacking. The purpose of this study was to investigate the outcome of a series of BD patients undergoing EVAR for aortic pseudoaneurysms.

## MATERIALS AND METHODS

### Clinical database and diagnostic criteria

With the approval of the institutional ethics committee, we conducted this retrospective study of the aortic aneurysm database at the Department of Vascular Surgery, Peking University People’s Hospital. We searched our center’s records of patients with aortic aneurysm undergoing surgery between January 2003 and September 2014 and we screened the enrolled cohort. Aortic pseudoaneurysm was confirmed by computed tomography angiography (CTA) and/or aortic angiography. The diagnosis of BD was confirmed according to the criteria described by the International Study Group for Behcet’s Disease. These criteria require the presence of recurrent oral ulceration in association with at least two of the following: characteristic ocular lesions, characteristic skin lesions, genital ulceration and a positive pathergy test result 12. Accordingly, 10 patients with BD undergoing open surgery or EVAR for aortic pseudoaneurysm constituted the final cohort.

The data were independently abstracted from medical records by two authors and were reviewed by the corresponding authors. The variables of interest included demographic characteristics, medical history and medications, laboratory test results, surgical records, postoperative medications and adverse events. Preoperative CTA was performed on all patients, not only to produce exact diagnostic evidence but also to provide an important reference for the individualized selection of an operative modality. Pseudoaneurysms whose outlets were located 2 cm below the outlets of the renal arteries were termed infra-renal, while those with outlets less than 2 cm below the outlets of the renal arteries were termed pararenal. Indications for endovascular or surgical intervention were acute symptoms including onset of worsening abdominal or back pain, organic dysfunction causing visceral hypoperfusion, unstable blood pressure or shock and any other evidence indicating aneurysmal rupture. Urgent indications such as acute onset of back and/or abdominal pain and abdominal tenderness on palpation with no signs of pseudoaneurysmal rupture on CT scans were established for surgical intervention within 24 hours 11.

### Technical surgical description

EVAR was the preferred modality for aortic pseudoaneurysm. Puncture for percutaneous access was performed through the right or bilateral common femoral artery under local anesthesia. The final decision concerning stent type and size was made by a surgeon according to the results of aortic angiography. This decision was also restricted based on the availability of a stent graft of the appropriate size at the time of the procedure. The extent of oversizing was typically 10% to 20% larger than the diameter of the aneurysmal neck diameter and was restricted to a maximum of 30% in urgent cases. Given the possibility of aortitis lesions near the pseudoaneurysm in vasculo-BD patients, for infra-renal aortic pseudoaneurysms, we placed the stent graft proximally exactly at the level of the inferior renal artery. A tubular stent graft was used for aneurysmal lesions located at the middle segment of the infra-renal abdominal aorta, whereas an aorto-uni-iliac or bifurcated stent graft was selected for aortic lesions near the aortic bifurcation while conducting an evaluation of the blood supply of the involved lower extremity. The stent grafts selected consisted of Zenith (Cook Medical, Bloomington, IN, USA), Endurant (Medtronic, Santa Rosa, CA, USA), Excluder (W.L. Gore & Associates, Flagstaff, AZ, USA) and Hercules-T (MicroPort Medical, Shanghai, China). The chimney technique and the multiple stent technique were adopted based on the individualized anatomy and the availability of stents for patients with juxtarenal or suprarenal pseudoaneurysms. Technical success was defined as the complete isolation of the aortic pseudoaneurysm without any type of endoleakage as confirmed by angiography.

As an alternative to EVAR, open surgery was performed under exceptional circumstances. General anesthesia was provided and all patients were managed in the intensive care unit post-operatively for one day. Prosthesis grafts with diameters similar to visually normal aortas were used. Interposition with end-to-end anastomosis was performed in the aorta and visceral reconstruction was conducted via end-to-end or end-to-side anastomosis. Aspirin at a dose of 100 mg per day was administered to patients undergoing aortic replacement, and dual antiplatelet therapy was administered to those undergoing visceral reconstruction. Technical success was defined as pseudoaneurysm removal and patency of visceral artery reconstruction.

### Medication strategy

Systemic immunosuppressive medication was administered under the suggestion of a rheumatic physician once BD was diagnosed. The erythrocyte sedimentation rate (ESR) and the C-reactive protein (CRP) level were used to estimate the status of rheumatic activity as a reference for medication adjustment. The normal ranges of ESR and CRP levels in our core laboratory were 0–15 mm/h (for males) and 0–8.00 mg/L, respectively.

### Follow-up evaluation

Follow-up was performed in March 2015. First, we contacted all enrolled patients by telephone and confirmed their survival status. Second, we recorded the date and cause of death for the non-surviving patients and we asked the surviving patients to visit for further consultation. Aortic ultrasound was used to identify aortic recurrence; aortic CTA was used as an alternative for those without contraindications, such as renal insufficiency. ESR and CRP levels were tested to evaluate rheumatic activity. Additionally, data regarding medication prescriptions by outpatient services, especially immunosuppressive, antiplatelet, anticoagulation medications, were reviewed in our center’s database for all enrolled patients.

### Statistical analysis

Quantitative variables were described as medians and extreme ranges and qualitative variables were expressed as numbers and percentages. Data were pooled and analyzed using SPSS version 19.0 (IBM SPSS, Chicago, IL). The patients were removed from the study upon death or loss to follow-up.

## RESULTS

### Patient characteristics

The final enrolled cohort was composed of 10 patients, all of whom were male. The median age was 41 years (from 29 years to 56 years). The most common chief complaint was durative abdominal or back dull pain, although 2 patients (20%) presented with abdominal masses. Six patients (60%) had extravascular damage, including 5 arterial aneurysm/pseudoaneurysm lesions, 3 arterial stenosis/occlusion lesions and 1 deep venous thrombosis. The renal artery was the most frequently involved artery (3 out of 10 patients, 30%) and other involved arteries included the superior mesenteric artery (SMA), common iliac artery (CIA), subclavian artery and coronary artery (Table 1).

Three patients had been diagnosed with BD prior to the manifestation of aneurysm-related symptoms under pre-operative systemic immunosuppressive medication. One patient was appropriately diagnosed with BD soon after admittance and thus received a high dose of prednisone before open surgery. The remaining 6 patients received systemic immunosuppressive medication after open surgery or EVAR once the diagnosis was confirmed. An elevated ESR was observed in 8 out of 9 patients, and an elevated CRP level was detected in all 9 patients. The median ESR and CRP level were 61 mm/h (from 10 mm/h to 105 mm/h) and 75 mg/L (from 19.6 mg/L to 421 mg/L), respectively. Prednisone was the initial systemic immunosuppressive medication for all patients and the initial dose was approximately 1 mg/kg per day. Cyclophosphamide was simultaneously administered to 5 patients and azathioprine to another 4 patients. One patient was prescribed adalimumab, a TNF-inhibiting anti-inflammatory drug.

**Figure 1:**
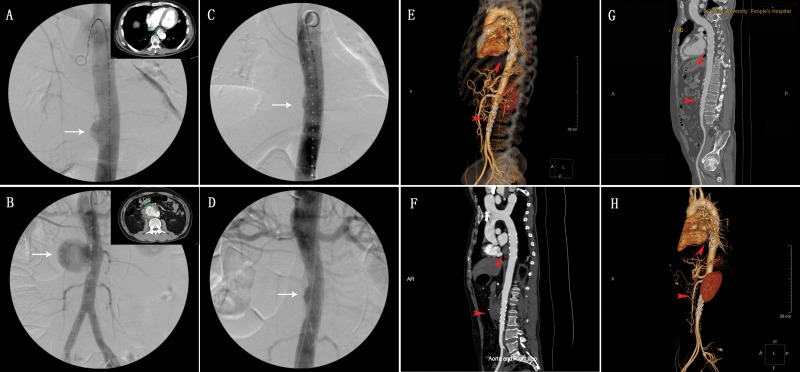
(A) Pararenal pseudoaneurysm and (B, C) infrarenal pseudoaneurysm.

In 7 patients, the outlet of the aortic pseudoaneurysm was located more than 2 cm below the outlet of the renal artery; in 2 patients, this outlet was located at the level of the renal artery outlet; and 1 patient, this outlet was located at the aorta between the SMA and renal artery (Figure 1). Additionally, the patients with infra-renal pseudoaneurysm had another pseudoaneurysm at the descending aorta and another patient with suprarenal pseudoaneurysm had multiple saccular aortic aneurysms at the descending aorta as well as the supra-renal and infra-renal abdominal aorta.

### Surgical outcome

**Figure 2:**
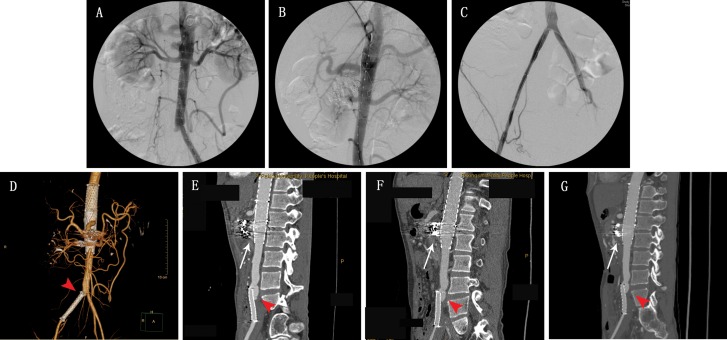
A 42-year-old male with descending thoracic and infrarenal abdominal aortic pseudoaneurysms (A, B). Aortogram following stent graft placement demonstrating that the aneurysm was successfully isolated (C, D). At the 1-week, 2-month, 6-month, and 12-month follow-up exams (E, F, G, H), three-dimensional volume rendering computed tomography (CT) images revealed that the aneurysm had completely regressed.

**Figure 3:**
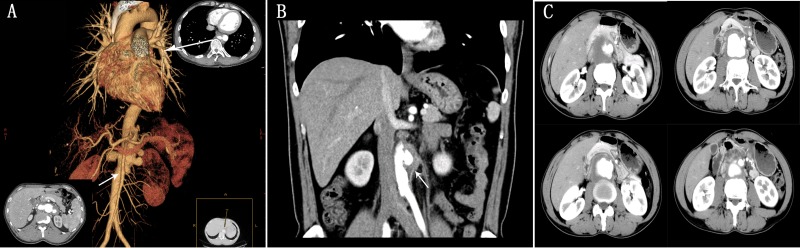
A 35-year-old male with suprarenal abdominal aortic pseudoaneurysm combined with right iliac arterial occlusion (A). Double- bare-metal stent placement combined with coiled embolization to treat the aortic lesion (B), following by balloon angioplasty and self-expansive bare-nitinol stent placement (C). At the 1-week follow-up, three-dimensional volume rendering CT showed obvious attenuation (D), and the lesion was barely detectable at the 2-month, 9-month and 15-month follow-up examinations (E, F, G). As indicated by the red arrow in the images below, a new aneurysm at the proximal end of the bare-metal stent of the iliac artery was detected at the 2-month follow-up examination and its size was stable in the subsequent follow-up examinations under close surveillance.

Eight patients underwent initial EVAR. 2 patients underwent initial open surgery: one patient exhibited shock due to acute pseudoaneurysmal rupture upon arrival at the emergency room and a stent graft was unavailable; the other patient had a challenging anatomy with involvement of the SMA and right renal artery. The overall technical success rate was 90% (9/10). The failed case was a pararenal aortic pseudoaneurysm accompanied by left renal arterial occlusion; the right renal artery and SMA were reconstructed via chimney graft deployment (self-expansive bare-nitinol stent). Type I endoleakage was identified on aortic angiography. The patient died due to rupture of the pseudoaneurysm 4 months after the initial EVAR. One patient requested additional thoracic EVAR for an accompanying thoracic aortic pseudoaneurysm (Figure 2) and another patient underwent percutaneous transluminal balloon angioplasty and stenting (self-expansive bare-nitinol stent) for combined right CIA occlusion the day after the initial EVAR using multilayer stenting and coiled embolization (Figure 3). Two urgent EVARs were performed on patients who suffered from rapidly worsening abdominal pain and technical success was achieved in both cases. No deaths occurred within 30 days after surgery.

### Follow-up

The median follow-up duration was 23 months (from 4 to 163 months, IQR 11 – 56 months). There were 7 recurrences in 5 patients (50%), consisting of 6 aortic recurrences and 1 femoral anastomotic recurrence. The median interval between previous operation and recurrence manifestation was 13 months (from 6 to 80 months, IQR 9–61.5 months). Moreover, the patient who underwent iliac stent deployment developed an iliac pseudoaneurysm proximally at the iliac stent, but the aortic pseudoaneurysm had disappeared 16 months after multiple bare-metal stents were placed for repair. The size of the new lesions was stable on the CTA imaging series and thus, no further intervention was performed.

Four aneurysm-related deaths occurred within the follow-up period. The overall 1-year, 3-year and 5-year survival rates were 80%, 64% and 48%, respectively. One of these patients was found to have type I endoleakage after EVAR, which required open surgery; however, because he exhibited multiple aortic aneurysms, thoracoabdominal aortic replacement was indicated. Finally, that patient refused further intervention and although immunosuppressive medication was administered, he died due to pseudoaneurysmal rupture. Three patients suffered from rupture of recurrent aortic pseudoaneurysms. Although one patient experienced sudden rupture of an undiscovered recurrent pseudoaneurysm, the other two patients requested careful follow-up instead of further surgery when recurrence was identified.

## DISCUSSION

In the present study, we retrospectively studied our experience in the treatment of BD patients with aortic pseudoaneurysms. Aortic pseudoaneurysm without surgical treatment had a very high mortality rate (up to 61%) due to pseudoaneurysmal rupture. Mulder et al. reported that the mortality rate of patients with non-infected pseudoaneurysms receiving nonsurgical treatment was up to 61% due to documented rupture; thus, the repair of all aortic pseudoaneurysms was strongly recommended 13. Considering that rupture of aortic pseudoaneurysms accounted for most deaths of these vasculo-BD patients, aggressive surgical interventions are indicated for all BD patients with aortic pseudoaneurysms.

The outcomes of our cohort confirmed the safety of EVAR for aortic pseudoaneurysm in BD patients, especially in emergency cases. There were no immediate complications from the stent graft procedures within 30 days after EVAR. Despite the controversy concerning the survival advantage of EVAR over open surgery for ruptured AAA 14, the excellent perioperative benefits of EVAR were convincing, as demonstrated in Veith’s study 8. Additionally, urgent open surgery was associated with many difficulties related to blood preparation, pre-anesthetic assessment and control of comorbidities, which lead to a high risk of perioperative complications 11,15,16. The widespread adoption of minimally invasive EVAR for aortic pseudoaneurysms in BD patient has been consistent with the trend in treatment for AAAs and its improvement in perioperative mortality was confirmed in our study 10,17-20.

However, complex AAA in BD patients, such as short infra-renal neck, juxtarenal or suprarenal locations, was not rare and the challenging anatomy of these patients limited endovascular repair and was associated with high morbidity and mortality. We observed that death related to failure of EVAR, predominantly in cases of complex AAA, was a predominant feature of postoperative short-term death. Yang reported that the only death in his cohort presented with leakage of a proximal anastomosis after repair of a ruptured AAA after undergoing stent-graft insertion. The patient ultimately died of ruptured pseudoaneurysm in the proximal and distal ends of the stent graft during the follow-up period 21. Similarly, the only failed procedure in our cohort occurred in a patient with a pararenal pseudoaneurysm. A fenestrated or branched endograft might be an alternative option for cases of technical failure 22; however, no branched endograft products were available in China and the fenestrated endograft used by Cook required a wait time of at least two weeks. For pseudoaneurysms with a complex anatomy in BD patients, effective and efficient surgical modalities remain under development and improvement.

We found that the frequency of post-operative recurrence was relatively high in our cohort. Open surgery had traditionally been the mainstream treatment for aortic pseudoaneurysms in BD patients, but the high rate of recurrence of this procedure troubled surgeons. Among the studies in which open surgery was performed on the majority of the study population, the rate of pseudoaneurysm recurrence varied from 22.4% to 47.6% 21,23,24. With the maturation of EVAR for AAA and the evidence of its benefit based on extensive studies, the rate of recurrence decreased to 10-20% in recent studies, in which most patients underwent EVAR as the initial intervention (Table 3)^10^,^20^,^25^-^27^. In our cohort, EVAR was the primary surgical modality, but the rate of recurrence in this study was similar to that of previous studies performing open surgery.

Further analysis demonstrated that the absence of systemic immunosuppressive medication prior to intervention in several patients might account for the abnormally high rate of recurrence in this study. The European League Against Rheumatism (EULAR) recommended that peripheral artery aneurysms require surgical repair accompanied by systemic immunosuppressive mediation [6]. Considering the studies reporting excellent outcome and a lack of recurrence, Liu and Kim stated that all patients in their studies received immunosuppressive medication to induce remission prior to intervention and that this treatment continued after intervention 20,27. Additionally, Kwon and Kim reported that only 1 patient in their cohort did not receive preoperative immunosuppressive medication due to a delayed diagnosis after intervention and exhibited co-morbid bilateral femoral head avascular necrosis, in which prednisone was contraindicated 10,26. In contrast, 6 out of 10 patients (60%) in our cohort had not received preoperative immunosuppressive medication, and 4 out of these 6 patients (66.7%) experienced recurrence. These patients did not receive systemic immunosuppressive treatment prior to EVAR because their diagnosis of BD was made after EVAR. Additionally, the preoperative ESR and CRP level in our cohort were clearly higher than those reported by Liu. Because ESR and CRP levels reflect the status of autoimmune activity, this observation indicated that the patients in our study were in an active stage of BD, which is strongly suspected to be associated with the high rate of recurrence in this study. Consequently, we believe that immunosuppressive medication was essential to defer the occurrence and development of recurrent aneurysms. However, further studies are needed to clarify related issues, including the necessary preoperative immunosuppressive treatment duration and regimen, the operative timing and the surveillance protocol.

The lessons from our study call attention to the co-morbidity of autoimmune disease with aortic pseudoaneurysm. The most common cause of aortic pseudoaneurysm was trauma, which was precisely diagnosed according to the medical history. However, concerning aortic pseudoaneurysm of unknown etiology, autoimmune disease should receive greater emphasis. The applied International Study Group (ISG) clinical diagnostic criteria for BD established in 1990 featured five specific syndromes for the diagnosis of BD: recurrent oral ulcerations, characteristic ocular lesions, characteristic skin lesions, genital ulcerations and positive pathergy test results 12. However, further studies showed that in 7-30% of these patients, vascular involvement was observed before the clinical diagnosis of BD 28,29. This delay in diagnosis led to a lack of prompt immunosuppressive medication for aortic pseudoaneurysm prior to intervention because of the urgency of surgical aortic repair to resolve the life-threatening risk to the patients. Fortunately the new International Criteria for BD (ICBD) published in 2014 included vascular manifestations as a class of clinical manifestations with diagnostic significance 30. Compared to the ISG diagnostic criteria, the ICBD was assumed to improve the prompt diagnosis of BD for patients with arterial involvement. In our study, neither the ESR or CRP level prior to operation nor the use of azathioprine or cyclophosphamide appeared to have a significant effect on recurrence. The proper duration and regimens of systemic immunosuppressive medication were not addressed in the EULAR recommendations 6. ESR and CRP levels serve as biomarkers for evaluating the status of BD activity. In our cohort, both azathioprine and cyclophosphamide were used and no significant difference in outcome between azathioprine and cyclophosphamide was observed. The optimal medical strategy will depend on additional evidence from multicenter large-scale controlled trials and this issue was beyond the scope of this study.

Our single-center study had a small sample size due to the low prevalence of BD patients with aortic pseudoaneurysm, and this small sample size rendered further univariate/multivariate analysis impossible. Additionally, over the wide time span (from 2003 to 2014) of the study, there was exciting and splendid progress in endovascular techniques; therefore, estimating the effect of these techniques among all patients may be somewhat unreasonable. However, our study remains of significance because of the current shortage of clinical experience in aortic pseudoaneurysms in BD patients; thus, the outcomes of our cohort together with the findings other published results by Kwon, Kim WH, Liu and Kim SW et al. could help to clarify the optimal surgical management for these patients 10,20,26,27.

Both open surgery and endovascular repair are safe and effective for treating aortic pseudoaneurysm in BD patients and these modalities directly decrease the high risk of death due to aortic pseudoaneurysmal rupture. However, effective immunosuppressive medication may be another essential factor in deferring the occurrence and development of recurrent aneurysms, although this conclusion requires additional evidence.

## AUTHOR CONTRIBUTIONS

Shen C and Zhang C conceived and designed the study. Li W, Zhang Y, Li Q and Jiao Y reviewed the manuscript and were responsible for the data collection. Li W and Zhang T were responsible for the data analysis. Shen C and Li W were responsible for the manuscript preparation and final approval. Shen C and Li W contributed equally to this work.

## Figures and Tables

**Table 1:** Demographic characteristics and follow-up data.

No.	Sex & Age	Symptoms and time of BD diagnosis	ESR prior to operation (mm/h)	CRP level prior to operation (mg/L)	Involved vessels beyond the aorta	Chief complaint	Duration from admittance to operation	Surgical treatment	Systemic immunosuppressive treatment	Duration of follow-up
1	M, 45	O, G, E Diagnosed after the second recurrence	61	75	-	Continuous back pain for 2 months and sudden exacerbation to unconsciousness within 2 hours	30 minutes	Aneurysmectomy reconstructed by aortic and bi-iliac artery interposition **Recurrence** at the proximal aortic anastomosis; underwent another aortic interposition 10 months after the first operation **Recurrence** again at the proximal aortic anastomosis and too close to renal artery to perform EVAR 8 months after the second operation	After rejection of surgery for the second recurrent pseudoaneurysm, prednisone qd 80 mg for 1 month followed by 50 mg for 6 months and 25 mg for 6 months, as well as cyclophosphamide q2w 400 mg for 3 months followed by q4w 400 mg for 3 months	30 months, dead (rupture of recurrent pseudoaneurysm at proximal aortic anastomosis)
2	M, 29	O, G, E1 day Diagnosed prior to the initial open surgery	105	421	Right renal artery pseudoaneurysm	Sudden tearing abdominal pain 15 days prior, followed by continuous dull pain	4 days	Prosthesis bypass from the aorta to the SMA, bilateral renal arteries, and bilateral CIA with aneurysmectomy	Pre-operative prednisone 40 mg once Post-operative prednisone qd 40 mg for 12 months followed by sequential reduction to drug withdrawal	58 months, alive (normal ESR and CRP level)
3	M, 45	O, E, S Diagnosed more than 6 years prior to the initial EVAR	-	-	-	Continuous abdominal pain combined with pulsatile abdominal mass for 1 month, acute enlargement of the mass for 1 day	3 hours	Infra-renal EVAR **Recurrence** at the proximal end of the endograft accompanied by right femoral anastomotic pseudoaneurysm 6 months after the initial EVAR, patient refused further intervention for aortic recurrence	Pre-operative prednisone for more than 6 years Post-operative prednisone qd 30 mg and azathioprine qd 100 mg for 7 months	7 months, dead (rupture of recurrent pseudoaneurysm at proximal aortic landing zone)
4	M, 33	O, G, S Diagnosed more than 3 years prior to the initial EVAR	10	62.8	Left subclavian aneurysm, Left renal arterial occlusion	Continuous dull epigastric pain for 1 month	6 days	EVAR with right renal artery and SMA chimney stenting **Technical failure** due to type I endoleakage	Pre-operative prednisone qd 40 mg, Cyclophosphamide q2w 400 mg for 36 months, prednisone qd 60 mg 1 month before operation and continued post-operatively	4 months, dead (rupture of aneurysm due to type Ia endoleak)
5	M, 28	O, G, S Diagnosed approximately 1 year prior to the first recurrence	26	19.6	Right renal artery stenosis	Continuous abdominal and back pain for 3 months	6 days	Infra-renal EVAR (Zenith, 22 mm/80 mm) **Recurrent** pseudoaneurysm at the distal end of the endograft, followed by another infra-renal EVAR (Aorto-bi-iliac) 80 months after the initial EVAR **Recurrent** pseudoaneurysm at the proximal end of the endograft above the right renal artery 13 months after the second EVAR; the size was stable based on close follow-up	Prednisone qd 30 mg for 12 months before the first recurrence, continued qd 30 mg post-operatively for another 12 months, qd 15 mg for half a year and qd 10 mg as a maintenance treatment	163 months, alive (regular hemodialysis owing to chronic kidney failure for 65 months)
6	M, 56	O, G, S Diagnosed after the initial EVAR	72	176	Left CIA pseudoaneurysm	Continuous pain in back and lower extremities for 2 months	2 days	Infra-renal EVAR (Medtronic, 25-14 mm /100 mm) combining artificial bypass from the right CFA to the left CFA and plugging of the left CIA**Rupture of recurrent aneurysm** 50 months after the initial operation	Prednisone qd 50 mg for 6 months followed by sequential reduction by 5 mg every two weeks after EVAR	50 months, dead (sudden death, rupture of recurrent aneurysm)
7	M, 39	O, G, E Diagnosed after the initial EVAR	45	24.2	-	Back pain for half a year and pulsatile abdominal mass for 20 days	5 days	Infra-renal EVAR (Hercules, 20-20 mm/60 mm) **Recurrent** pseudoaneurysm at the distal end of the endograft, followed by another infra-renal EVAR(Aorto-bi-iliac) 73 months after the initial EVAR	Prednisone and azathioprine for approximately 3 years after the first EVAR, leflunomide qd 20 mg for 12 months after the second EVAR	108 months, alive (normal ESR of 8 mm/h and CRP level of 6.29 mg/L)
8	M, 35	O, G, P Diagnosed after the initial EVAR	30	66.8	Right CIA occlusion, SMA aneurysm	Intermittent epigastric pain for more than 1 year	2 days	Juxta-renal EVAR (Sinus XL. 22 mm/80 mm, 24 mm/80 mm) combining coil embolization and PTA stenting for the R-CIA	Prednisone qd 50 mg for 6 months followed by sequential reduction by 5 mg every two weeks, Cyclophosphamide q2w 400 mg for 16 months after the initial intervention	16 months, alive (normal ESR of 3 mm/h and slightly elevated CRP level of 11.20 mg/L, new aneurysm at the proximal landing zone of the bare-metal iliac stent)
9	M, 42	O, G, S Diagnosed 3 years prior to the initial EVAR	98	285	Coronary artery circumflex aneurysm, deep venous thrombosis	Intermittent thoracic pain for more than 2 years and abdominal pain for 3 months	1 day	TEVAR (Hercules, 26 mm/80 mm) and infra-renal EVAR (Hercules, 18 mm/80 mm)	Pre-operative prednisone qd 50 mg for 10 months followed by 25 mg for 12 months, 15 mg for 3 months and 7.5 mg for 11 months, as well as Cyclophosphamide q2w 400 mg for 24 monthsPost-operative adalimumab q2w 40 mg combined with prednisone qd 5 mg for 12 months followed by prednisone qd 20 mg and azathioprine 150 mg for 4 months	16 months, alive (normal ESR of 10 mm/h and CRP level of 1.50 mg/L)
10	M, 46	O, G, E Diagnosed after the initial EVAR	74	88	-	Abdominal and back pain for over 10 days, acute deterioration for 12 hours	1 hour	Infra-renal EVAR (Hercules, 18 mm/80 mm)	Post-operative prednisone 30 mg qd for 3 months, and increase to 50 mg up to present, azathioprine 100 mg bid up to present	9 months, alive (normal ESR of 13 mm/h and CRP level of 3.32 mg/L)

Abbreviations: O: ocular lesions; S: skin lesions; G: genital ulceration; E: uveitis; P: positive pathergy test result.

**Table 2:** Summary of the published studies on open surgery and EVAR for aneurysmal lesions in patients with BD.

First author	Published year	Total no. of cases	Involvement	Number of open heart surgeries	Number of EVAR procedures	Number of aneurysm-related deaths	Number of recurrences	Systemic immunosuppressive medication	Follow-up (months)
Kwon	2008	12	Abdominal aortic aneurysm	21	0	1 / 12(rupture of recurrent aneurysm)	8//21 (38.1%)	Only postoperative medication (steroids, colchicine, azathioprine, or cyclophosphamide)was administered to all patients	45.4
Tuzun	2012	25	Peripheral arterial aneurysm	22	0	1/22(anastomotic dehiscence)	5/22 (22.7%)	Immunosuppression with cyclophosphamideand corticosteroids before intervention and continued post-operatively	88.8
Yang	2013	21	Peripheral arterial lesions	24	10	1/21(leakage after EVAR and consequent rupture of pseudoaneurysm)	10/21 (47.6%)	Combination of medications including azathioprine, steroids and colchicine before intervention except in 10 patients who had been referred from other institutes	78.7
Park	2001	7	Peripheral arterial aneurysm	0	9	0	1/7 (14.3%)	Not described.	28
Kwon	2003	9	Arterial pseudoaneurysm	0	11	0	1/9 (11.1%)	Immunosuppressive agents (azathioprine,prednisolone) before and after the procedure except in 1 patient with a delayed diagnosis	24.1
Kim WH	2009	34	Non-cerebral arterial aneurysm	7	16	1/23(aneurysm-related death after EVAR)	4/23 (17.4%)	Prednisolone 60 mg/d before intervention to induce remission	47.6
Liu	2009	10	Aortic pseudoaneurysm	0	10	1/10(rupture of recurrent pseudoaneurysm)	2/10 (20%)	All patients received immunosuppressive therapy before and after intervention	21.5
Kim SW	2014	10	Aortic pseudoaneurysm	0	10	0	1/10 (10%)	Immunosuppressivetherapy at the time of EVAR and continued during follow-up except in 1 patient	57
